# Retraction: Naringin protects endothelial cells from apoptosis and inflammation by regulating the Hippo-YAP Pathway

**DOI:** 10.1042/BSR-2019-3431_RET

**Published:** 2024-10-07

**Keywords:** apoptosis, Endothelial cells, Hippo-YAP Pathway, inflammation, Naringin

This Retraction follows a Correction relating to this article previously published by Portland Press.

This article “Naringin protects endothelial cells from apoptosis and inflammation by regulating the Hippo-YAP Pathway” DOI: 10.1042/BSR20193431 is being retracted from *Bioscience Reports* at the request of the Editor-in-Chief and the Editorial Board. This follows receipt of a notification from a reader, alerting the Editorial Board to multiple similarities between data in this paper and that present in other articles by different authors. Specifically:

- Figure 1C which seems to appear as Figure 1A from Zhu et al, 2020 (DOI: 10.21037/tau.2020.03.11) with panel rearrangement
- Figure 2A which seems to appear as Figure 1D from Yan et al, 2017 (DOI: 10.3892/ijo.2017.4216) [retracted] with panel rearrangement and modification, and part of which seems to appear in Figure 7E from Zhang et al, 2018 (DOI: 10.3892/ijo.2018.4586) [retracted] with colour alteration
- Figure 2C in the Correction which seems to appear as Figure 1E from Sun et al, 2018 (DOI: 10.12659/msm.908927) with panel rearrangement
- Figure 3A and 3B that seems to appear as Figure 4D and 4E from Chen et al, 2019 (DOI: 10.7150/ijbs.30193)
- Figure 4C which seems to appear as Figure 4B from Sun et al, 2018 (DOI: 10.12659/msm.908927) with panel rearrangement, and separately part of which seems to appear in Figure 4a from Yu et al, 2022 (DOI: 10.1080/21655979.2022.2081463).

Additionally, the Editorial Team has concerns surrounding potential signs of image manipulation in Figure 2A ox-LDL 80 µg/ml Naringenin 100 µM panel. The authors have been contacted with regards to the retraction and have not responded to the Journal's queries or the concerns raised. Given the extent of the issues raised, the Editorial Board stand by the decision to retract the article.

